# PDE4B Induces Epithelial-to-Mesenchymal Transition in Bladder Cancer Cells and Is Transcriptionally Suppressed by CBX7

**DOI:** 10.3389/fcell.2021.783050

**Published:** 2021-12-16

**Authors:** Zhengnan Huang, Jiakuan Liu, Jiale Yang, Yilin Yan, Chenkai Yang, Xiao He, Ruimin Huang, Mingyue Tan, Denglong Wu, Jun Yan, Bing Shen

**Affiliations:** ^1^ Department of Urology, Tongji Hospital, Tongji University School of Medicine, Shanghai, China; ^2^ Department of Laboratory Animal Science, Fudan University, Shanghai, China; ^3^ Shanghai Institute of Materia Medica, Chinese Academy of Sciences, Shanghai, China; ^4^ School of Pharmacy, University of Chinese Academy of Sciences, Beijing, China; ^5^ Department of Urology, Shanghai General Hospital, Shanghai Jiaotong University School of Medicine, Shanghai, China; ^6^ School of Chinese Materia Medica, Nanjing University of Chinese Medicine, Nanjing, China; ^7^ Department of Urology, Shuguang Hospital, Shanghai University of Traditional Chinese Medicine, Shanghai, China

**Keywords:** phosphodiesterase 4B, urinary bladder cancer, chromobox protein homolog 7, epithelial-to-mesenchymal transition, β-catenin

## Abstract

Urinary bladder cancer (UBC) is a common malignant tumor with high incidence. Advances in the diagnosis and treatment of this disease demand the identification of novel therapeutic targets. Multiple studies demonstrated that PDE4B level was upregulated in malignancies and high PDE4B expression was correlated with poor outcomes. Herein, we identified that PDE4B was a potential therapeutic target in UBC. We confirmed that PDE4B expression was correlated with aggressive clinicopathological characteristics and unfavorable prognosis. Functional studies demonstrated that ectopic expression of PDE4B promoted UBC cells proliferation, migration and invasion, whereas PDE4B depletion suppressed cancer cell aggressiveness. We also identified CBX7 as a regulator of PDE4B to suppress the expression of PDE4B at the transcription level in a PRC1-dependent manner. Moreover, our results indicated that PDE4B induced epithelial-to-mesenchymal transition (EMT) in UBC cells *via* β-catenin pathway, whereas inhibition of PDE4B by its small molecule inhibitor, rolipram, effectively reversed the PDE4B overexpression-induced effects. To sum up, our results indicated that PDE4B acts as an oncogene by promoting UBC cell migration and invasion *via* β-catenin/EMT pathway.

## Introduction

Urinary bladder cancer (UBC) is the fourth most common malignant tumor in men and a common malignant tumor in women (Siegel et al., 2021). There are estimated 500,000 new cases and 200,000 deaths of UBC worldwide (Lenis et al., 2020). Although significant progress has been made in surgery and drug treatment in UBC, the recurrence and metastasis rate remain high (Antoni et al., 2017). Therefore, advances in understanding of the underlying molecular mechanisms of UBC are urgent for us to diagnose and treat this disease more accurately.

Epithelial-to-mesenchymal transition (EMT) process occurs in various physiological and pathological conditions and has been implicated in cancer metastasis, cancer stemness maintenance, and chemoresistance (van der Horst et al., 2012; Dongre and Weinberg, 2019). In UBC patients, the high EMT score based on RNAseq data is strongly associated with UBC patients with poor prognosis (McConkey et al., 2016; Robertson et al., 2017). As one of the well-known EMT inducer, the activation of TGFβ/Smad signaling by paracrine TGFβ1 from cancer-associated fibroblasts increases EMT-associated transcription factors, including SNAIL and ZEB2, and represses epithelial marker, E-Cadherin, in UBC cells (Zhuang et al., 2015; Zhuang et al., 2017). In addition, the constitutive activations of WNT/β-catenin and Hippo signaling pathways have been recently reported to promote EMT and UBC cell invasion, *via* the overexpression of Wnt ligand Wnt7A, and transcriptional factor TEAD4 (Huang et al., 2018; Huang et al., 2021a). However, there may exist altered signaling pathways other than the aforementioned pathways in EMT process.

Phosphodiesterase 4B (PDE4B) is one of the members of the phosphodiesterase (PDE) family, whose function is to decompose cyclic nucleotides, such as cAMP and cGMP, thereby reducing the signal transduction of these important second messengers in cells (Soderling and Beavo, 2000). The PDE superfamily is subdivided into 11 families, among which, the cAMP-specific PDE4B is crucial in regulating intracellular signal transduction, including activation of PKA and extracellular signal-regulated kinases, as well as G protein conversion and regulation of receptor desensitization (Skalhegg and Tasken, 2000; Conti et al., 2003; Houslay and Adams, 2003). The dysregulation of PDE4B is involved in a wide range of diseases progression, including Parkinson’s disease, depression, asthma, chronic obtrusive pulmonary disease, and cancer (Houslay et al., 2005; Zhang et al., 2005; Fan Chung, 2006). Interestingly, the function of PDE4B is cancer-type dependent, i.e., PDE4B overexpression increases malignancy in diffuse large B-cell lymphoma and non-small cell lung cancer (Smith et al., 2005; He et al., 2017), while its underexpression contributes to prostate cancer progression. Hence, it is worth characterizing the exact role of PDE4B in UBC development, which has not been clearly elucidated.

Herein, we firstly investigated the correlation of PDE4B expression with clinicopathological characteristics and prognosis. Next, functional studies and bioinformatics analysis were carried out to define the role of PDE4B in EMT of UBC cells and cancer aggressiveness. Interestingly, we also identified CBX7 as an upstream regulator of PDE4B, which inhibited PDE4B expression by means of transcriptional repression. At last, we examined the effects of the inhibition of PDE4B activity by its small molecule inhibitor Rolipram.

## Materials and Methods

### Cell Culture and Reagents

UBC cell lines (5637, SW780, T24, UMUC-3, and SCaBER) and nonmalignant urothelial cell line (SV-HUC-1) were cultured in RPMI 1640 medium (Life Technologies) supplemented with penicillin/streptomycin (Invitrogen, United States) and 10% FBS at 37°C with 5% CO_2_. Reagents and chemicals used in this study were listed in Supplementary Table S1.

### Clinical Samples

Formalin-fixed, paraffin-embedded UBC tissues and relevant clinicopathological variables were acquired from Shanghai General Hospital. The informed consent of all patients was obtained, and the research protocol was formally approved by the ethics committee of Shanghai General Hospital.

### Bioinformatics Analysis

Gene expression and clinicopathologic information of The Cancer Genome Atlas Urothelial Bladder Carcinoma (TCGA-BLCA) were downloaded from Genomic Data Commons Data Portal. Other public microarray data were retrieved from the Gene Expression Omnibus (GEO) database.

### Cell Line Establishment

The PDE4B and CBX7 overexpressing cell lines were obtained by infecting with virus expressing Flag-tagged PDE4B (pCDH-3xFlag-PDE4B) and CBX7 (pCDH-3xFlag-CBX7), respectively. For knockdown assays, two siRNA targeting PDE4B were transfected into cells using Lipofectamine 3000 (Thermo Fisher Scientific). Two CBX7 shRNA were transfected into cells using the lentiviral pLKO.1 backbone. The siRNA and shRNA sequences used were listed in Supplementary Table S2.

### Western Blotting

Total protein was isolated by lysis buffer with protease and phosphatase inhibitor cocktail (Roche, Basel, Switzerland). Western blotting was performed following the standard methods. Antibodies used in this study were listed in Supplementary Table S3.

### MTT Assay

2,000 cells/well were seeded into a 96-well plate. If cells adhered to the bottom, 10 μL MTT [3-(4,5-dimethylthiazol-2-yl)-2,5-diphenyltetrazoliumbromide] was added to each well for 4 h at 37°C and it was identified as 0 h. The formazan crystals were dissolved in dimethyl sulfoxide (DMSO) at 37°C for 15 min and the absorbance at 490 nm was examined. After 24, 48, and 72 h, the similar procedure was carried out.

### Wound Healing Assay

Confluent cultures in 6-well plates were scratched with a 200 μL pipette tip, washed with PBS and then cultured in medium containing 1% FBS. Images were captured after 0, 24, and 48 h respectively to estimate wound closure (%).

### Transwell Invasion Assay

Cell invasion was measured using 8 μm pore-size transwell inserts (Millipore, United States). 1 × 10^5^ cells were seeded into the top chamber pre-coated with 1:8 diluted Matrigel, then 500 μL medium containing 10% FBS was added to the bottom chamber. After the incubation for 24 h, cells were fixed in formaldehyde, stained with crystal violet and counted.

### Gene Set Enrichment Analysis

GSEA software was applied to explore the correlation of PDE4B expression on the progression of UBC. Gene set collections were downloaded from MSigDB database. Nominal *p*-value <0.05 and FDR <0.25 were considered to a statistically significant gene set.

### Immunohistochemistry

5 μm thick paraffin-embedded bladder tumor sections were prepared and stained according to the previously described procedure (Yan et al., 2020). Antibodies used were listed in Supplementary Table S3.

### Chromatin Immunoprecipitation

The ChIP assay was performed as previously described (Chang et al., 2018). Briefly, cells were fixed in 1% paraformaldehyde for 10 min at room temperature, following quenched using 0.125 mol/L glycine for 5 min. The formaldehyde-fixed cells pellets were lysed with cell lysis buffer and sonicated using Diagenode Bioruptor Pico Sonicator for 30 cycles (30 s/90 s on/off) to obtain a DNA range of 200–1,000 bp. Samples were incubated with protein A/G (Millipore, #17-371) and antibody (anti-CBX7, Abcam, ab21873; anti-ubH2AK119, Cell Signaling Technology, #8240S) overnight at 4°C. Samples were washed 4 times with low salt, high salt, LiCl, and TE wash buffers, respectively. Elution of protein/DNA complexes were reversed cross-link to obtain free DNA at 62°C for 2 h with shaking and incubated at 95°C for 10 min. DNA was purified using spin columns (Millipore, #17-10085). Individual ChIP samples were analyzed by qRT-PCR using PED4B promoter primers. The primers used were listed in Supplementary Table S2.

### Statistical Analysis

All statistical analyses were performed using GraphPad Prism 8.0. Data were presented as means ± standard deviation (SD). Student’s *t* test was performed to compare statistical significance between groups. Pearson correlation was used to analyze the association between genes expression. A chi-square test was employed to assess the association between PDE4B expression and clinicopathological variables. Kaplan-Meier (Log rank test) method was performed to estimate the overall survival. A value of *p* < 0.05 was considered statistically significant.

## Results

### The Association of PDE4B and Clinicopathological Characteristics

**FIGURE 1:**
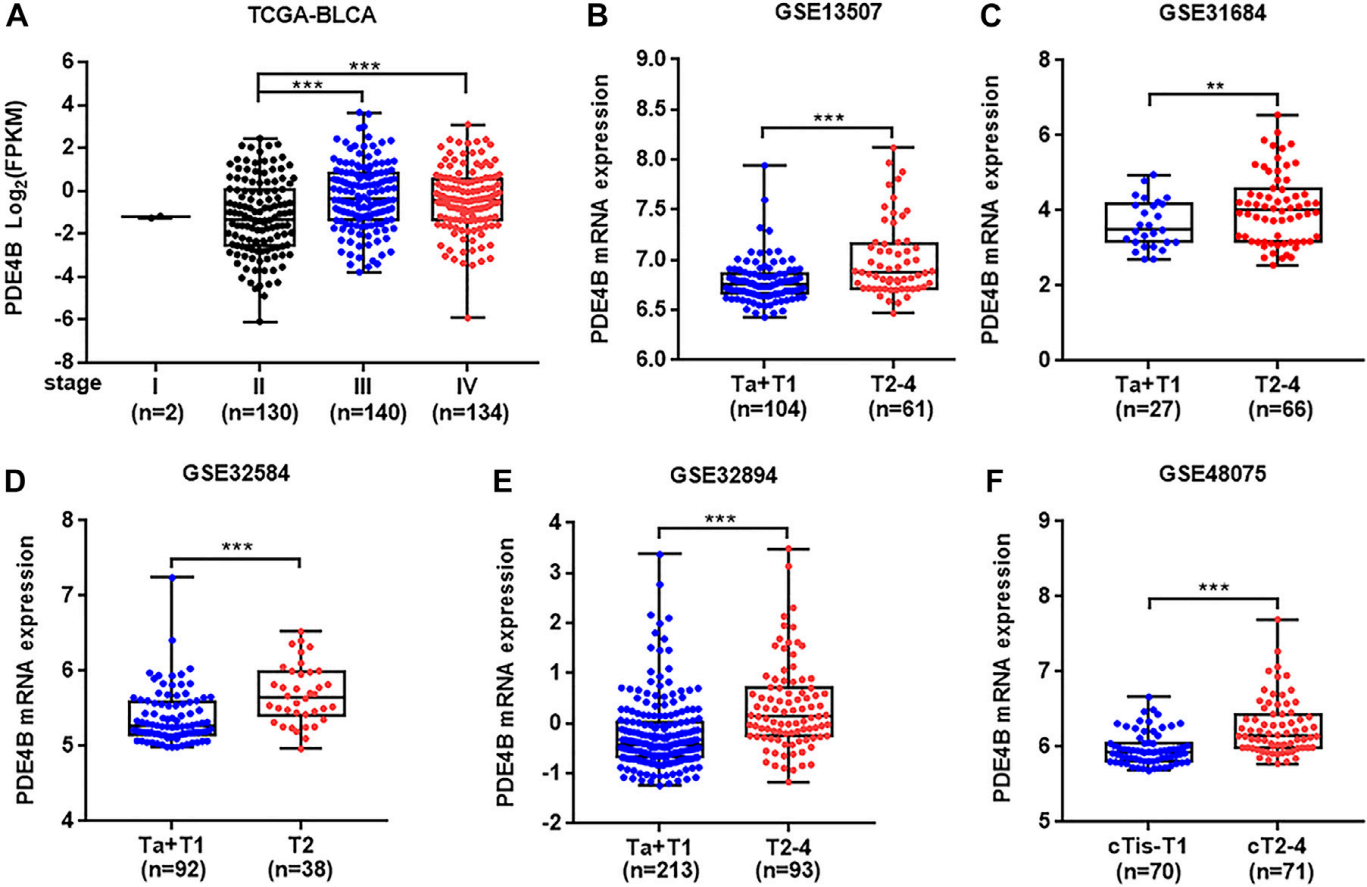
PDE4B expression is upregulated in advanced stage patients with UBC. **(A–F)** The comparison of PDE4B mRNA level in different tumor stage of UBC patients from TCGA-BLCA **(A)**, GSE13507 **(B)**, GSE31684 **(C)**, GSE32584 **(D)**, GSE32894 **(E)**, and GSE48075 **(F)** databases. **p < 0.01, ***p < 0.001.

**FIGURE 2:**
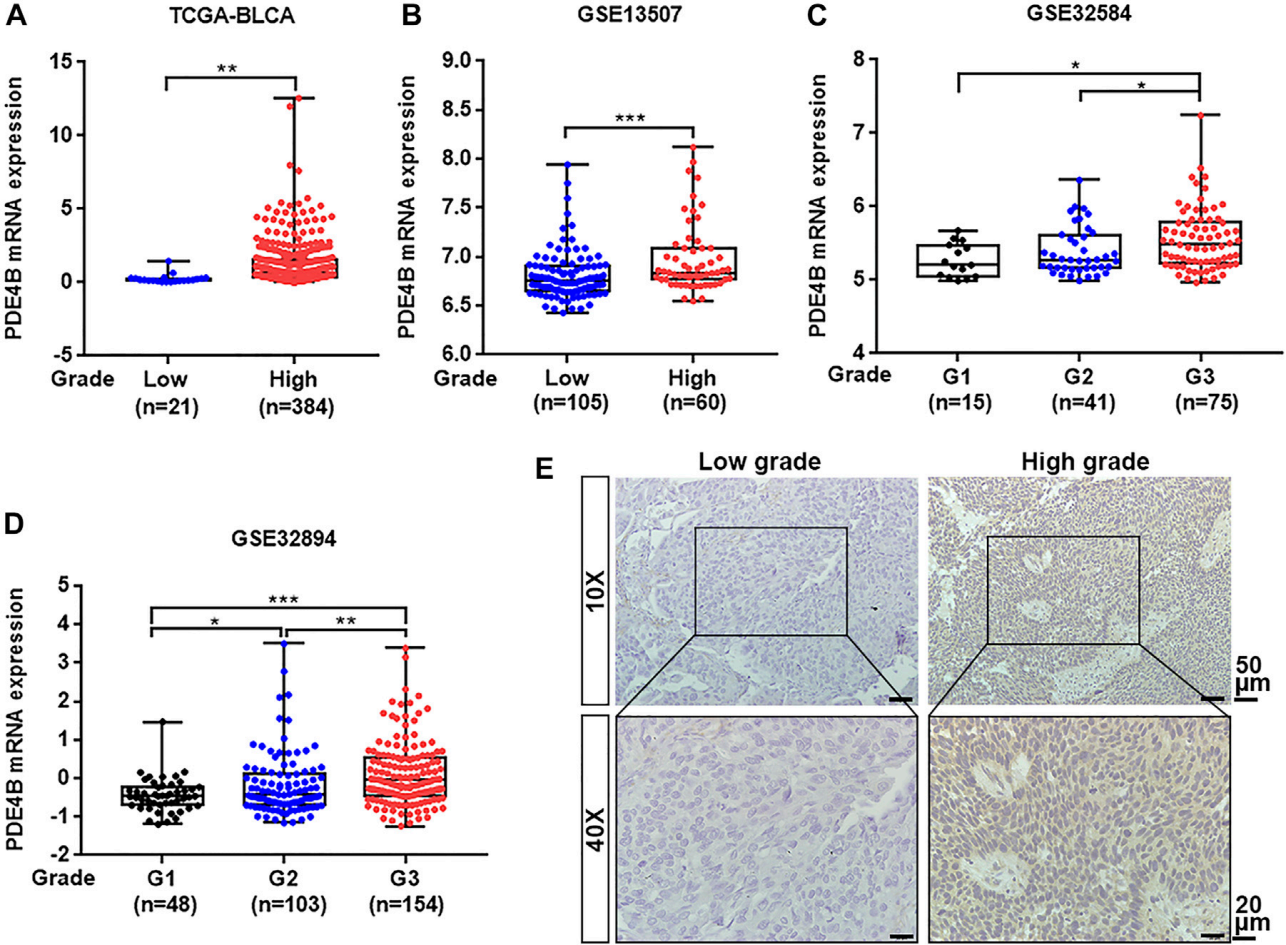
PDE4B expression is elevated in high-grade patients with UBC. **(A–D)** The comparison of PDE4B mRNA level in low-grade and high-grade UBC patients from TCGA-BLCA **(A)**, GSE13507 **(B)**, GSE32584 **(C)**, and GSE32894 **(D)**. **(E)** Representative IHC staining images of the PDE4B expression in low-grade and high-grade UBC tissues. Scale bar, 50 and 20 μm *p < 0.05, **p < 0.01, ***p < 0.001.

To investigate whether PDE4B was involved in UBC development, multiple databases containing tumor clinicopathological information were analyzed. As shown in Figures 1A–F, higher PDE4B mRNA level was observed in advanced stages patients from TCGA-BLCA and GEO databases (*p* < 0.01). Moreover, PDE4B mRNA level was elevated in high-grade patients than that in low-grade patients (*p* < 0.05, Figures 2A–D). Consistently, our IHC data also showed that high-grade patients expressed higher level of PDE4B protein compared to low-grade patients (Figure 2E). In addition, PDE4B protein level was significantly associated with tumor grade (*p* = 0.036; Table 1) and T stage (*p* = 0.003; Table 1). In summary, we concluded that the PDE4B expression was closely correlated with clinicopathological characteristics.

**TABLE 1:** The association between PDE4B protein levels and clinicopathological features of UBC patients (*n* = 59).

Characteristics	Number	Expression of PDE4B		*p* value
		High (n, %)	Low (n, %)	
Gender				0.173
male	54	26 (48.1%)	28 (51.9%)	
female	5	4 (80.0%)	1 (20.0%)	
Age				0.711
≥ 60	42	22 (52.4%)	20 (47.6%)	
< 60	17	8 (47.1%)	9 (52.9%)	
Tumor grade				**0.036**
Low	17	5 (29.4%)	12 (70.6%)	
High	42	25 (59.5%)	17 (40.5%)	
T stage				**0.003**
Ta-1	31	10 (32.3%)	21 (67.7%)	
T2-4	28	20 (71.4%)	8 (28.6%)	
N stage				0.081
N0	56	27 (48.2%)	29 (51.8%)	
≥N1	3	3 (100.0%)	0 (0.0%)	
M stage				**0.023**
M0	48	21 (43.8%)	27 (56.2%)	
≥ M1	11	9 (81.8%)	2 (18.2%)	

Numbers in bold indicate *p* value with statistical significance.

### PDE4B Overexpression Predicts Poor Prognosis in UBC Patients

**FIGURE 3:**
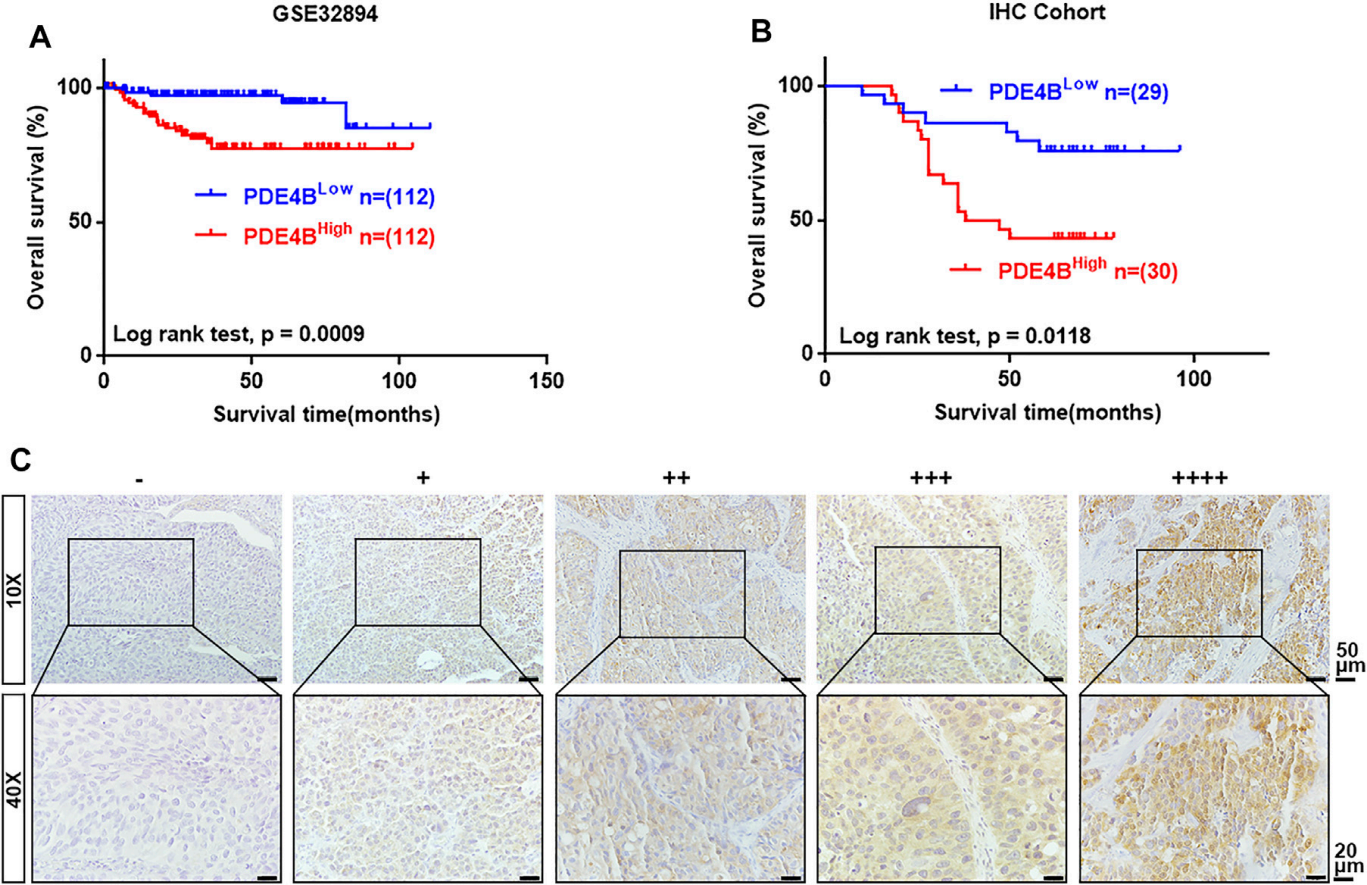
The upregulated level of PDE4B correlates with unfavorable prognosis in UBC patients. **(A, B)** Kaplan–Meier plot of overall survival of UBC patients from GSE32894 **(A)** and IHC cohort **(B)**. **(C)** Representative IHC staining images of different PDE4B staining intensities in UBC tissues. Scale bar, 50 and 20 μm.

Next, we performed Kaplan-Meier survival analysis to examine the clinical value of PDE4B in UBC. In GSE32894 database, higher PDE4B mRNA expression heralded worse prognosis (*p* = 0.0009, Figure 3A). To validate the results from public database, we collected 59 UBC specimens for IHC and made survival analysis. Consistently, higher PDE4B protein level indicated shorter overall survival time (*p* = 0.0118, Figure 3B). The representative images of IHC staining of different PDE4B staining intensities were displayed in Figure 3C. Collectively, these findings revealed that upregulation of PDE4B was remarkably correlated with unfavorable prognosis in patients with UBC.

### Ectopic PDE4B Promotes UBC Cells Proliferation and Invasion

**FIGURE 4:**
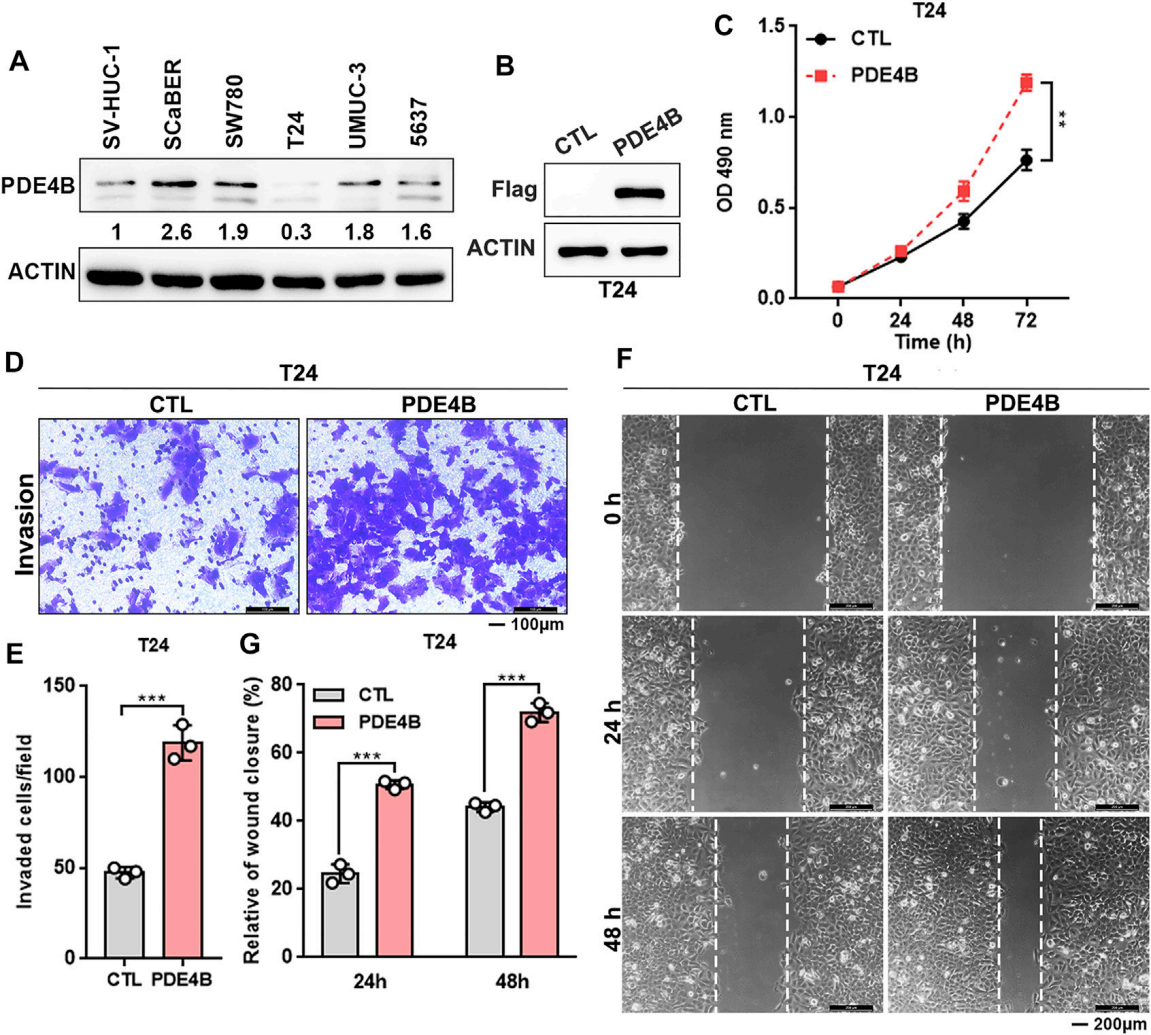
Ectopic PDE4B promotes UBC cells proliferation and invasion. **(A)** Endogenous expression of PDE4B in multiple cell lines were detected by western blotting. **(B)** Ectopic expression of PDE4B in T24 cell was confirmed by western blotting. **(C)** Effects of PDE4B overexpression on cell viability. **(D–G)** Effects of PDE4B overexpression on cell invasion and migration examined by transwell **(D,E)** and wound healing **(F,G)** assays. Scale bar, 100 μm **(D)** and 200 μm **(F)**. **p < 0.01, ***p < 0.001.

Above data indicated that PDE4B may function as an oncogene in UBC progression. Furthermore, we found that most UBC cell lines expressed higher PDE4B level compared with nonmalignant urothelial cell line SV-HUC-1 (Figure 4A and Supplementary Figure S1). Interestingly, we also observed that higher expression of PDE4B was specifically in basal/squamous subtype, which was the UBC subtypes with poor prognosis (Supplementary Figure S2). To investigate potential oncogenic role of PDE4B, we stably ectopically expressed PDE4B in T24 cells, which expressed relatively lower endogenous PDE4B (Figures 4A,B). Firstly, we explored the effects of PDE4B on cell proliferation. The results showed that ectopic expression of PDE4B notably promoted T24 cells viability (Figure 4C). In line with this, overexpression of PDE4B markedly enhanced cancer cells invasiveness (Figures 4D,E). Wound healing assays also demonstrated that cells overexpressing PDE4B significantly facilitated cell migration (Figures 4F,G). Taken together, these results indicated that overexpression of PDE4B facilitated UBC cells proliferation and invasion.

### Knockdown of PDE4B Inhibits UBC Cells Proliferation and Invasion

**FIGURE 5:**
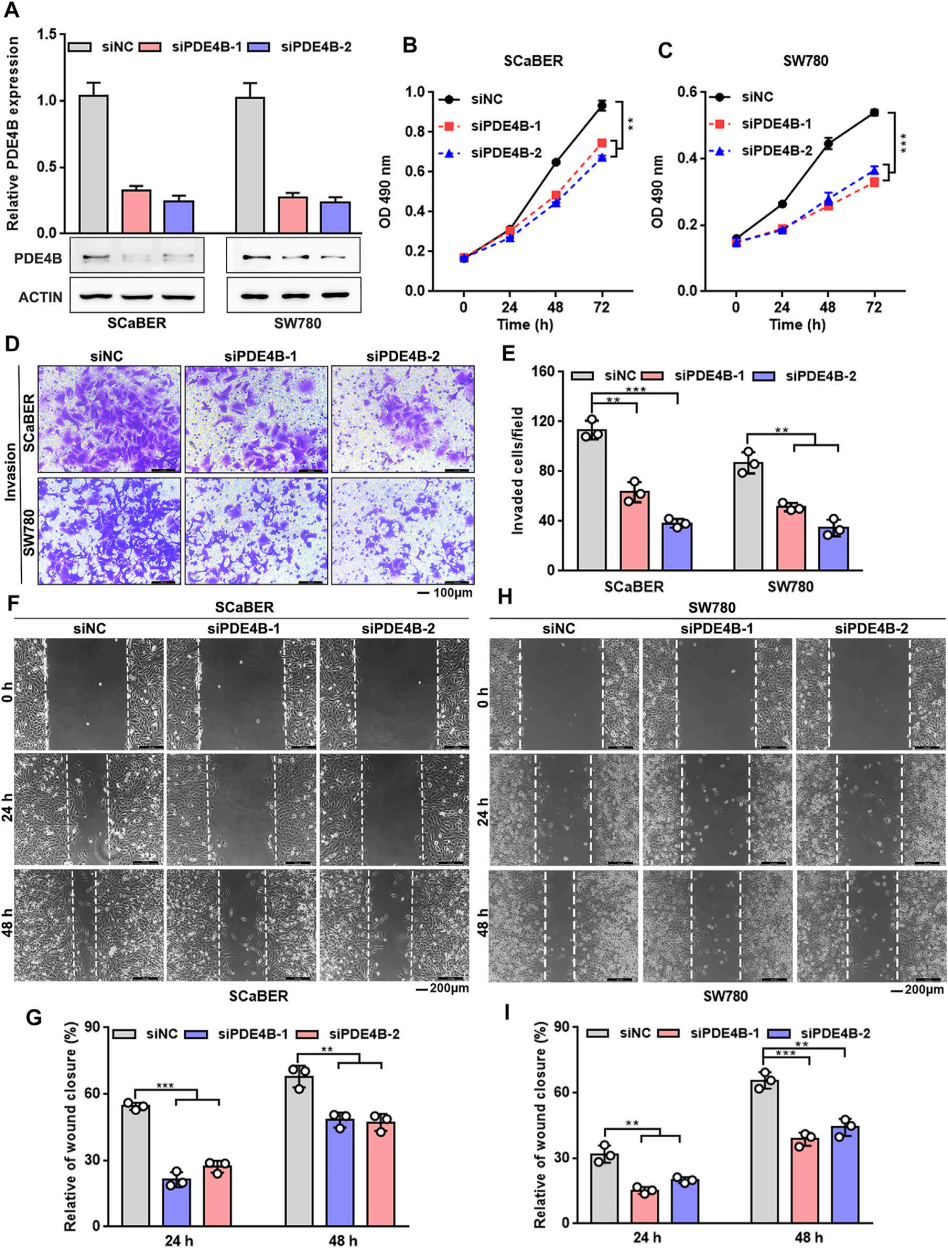
PDE4B knockdown inhibits UBC cells proliferation and invasion. **(A)** PDE4B knockdown in SCaBER and SW780 cells were confirmed by qRT-PCR and western blotting after transfection with siRNAs. **(B,C)** Effects of PDE4B knockdown on cell viability. **(D–I)** Effects of PDE4B knockdown on cell invasion and migration examined by transwell **(D,E)** and wound healing **(F–I)** assays. Scale bar, 100 μm **(D)** and 200 μm **(F,H)**. **p < 0.01, ***p < 0.001.

We then conducted the loss-of-function approach to further explore the function of PDE4B. SCaBER and SW780 cells were selected for knockdown as the relatively higher level of endogenous PDE4B protein (Figure 4A). PDE4B expression was decreased after two independent siRNAs to PDE4B were transfected into cells (Figure 5A). As shown in Figures 5B,C, knockdown of PDE4B markedly inhibited SCaBER and SW780 cells viability. Consistently, PDE4B deficiency notably suppressed cells invasive abilities (Figures 5D,E). Wound healing also demonstrated that PDE4B-silenced SCaBER and SW780 cells filled the gap much slowly than control cells (Figures 5F–I). Taken together, these functional data implied that depletion of PDE4B reduced the capability of UBC cells to proliferate and invade.

### CBX7 Transcriptionally Represses PDE4B in a PRC1-Dependent Manner

The Chromobox protein homolog 7 (CBX7) belongs to the Polycomb Group (PcG) family, which is a component of canonical polycomb repressive complex1 (PRC1), contributing to transcriptional target genes repression by prompting the RING protein in PRC1 to monoubiquitinate H2AK119 (Simon and Kingston, 2009; Musselman et al., 2012). In our previous study, downregulation of PDE4B expression was observed in CBX7 knockdown cells. When we looked up the RNAseq dataset (GSE158224) in our previous study, we observed that PDE4B was downregulated in CBX7 overexpressing cells, compared to control T24 cell group (Huang et al., 2021b). Thus, we speculated that CBX7 may be a potential regulator of PDE4B.

**FIGURE 6:**
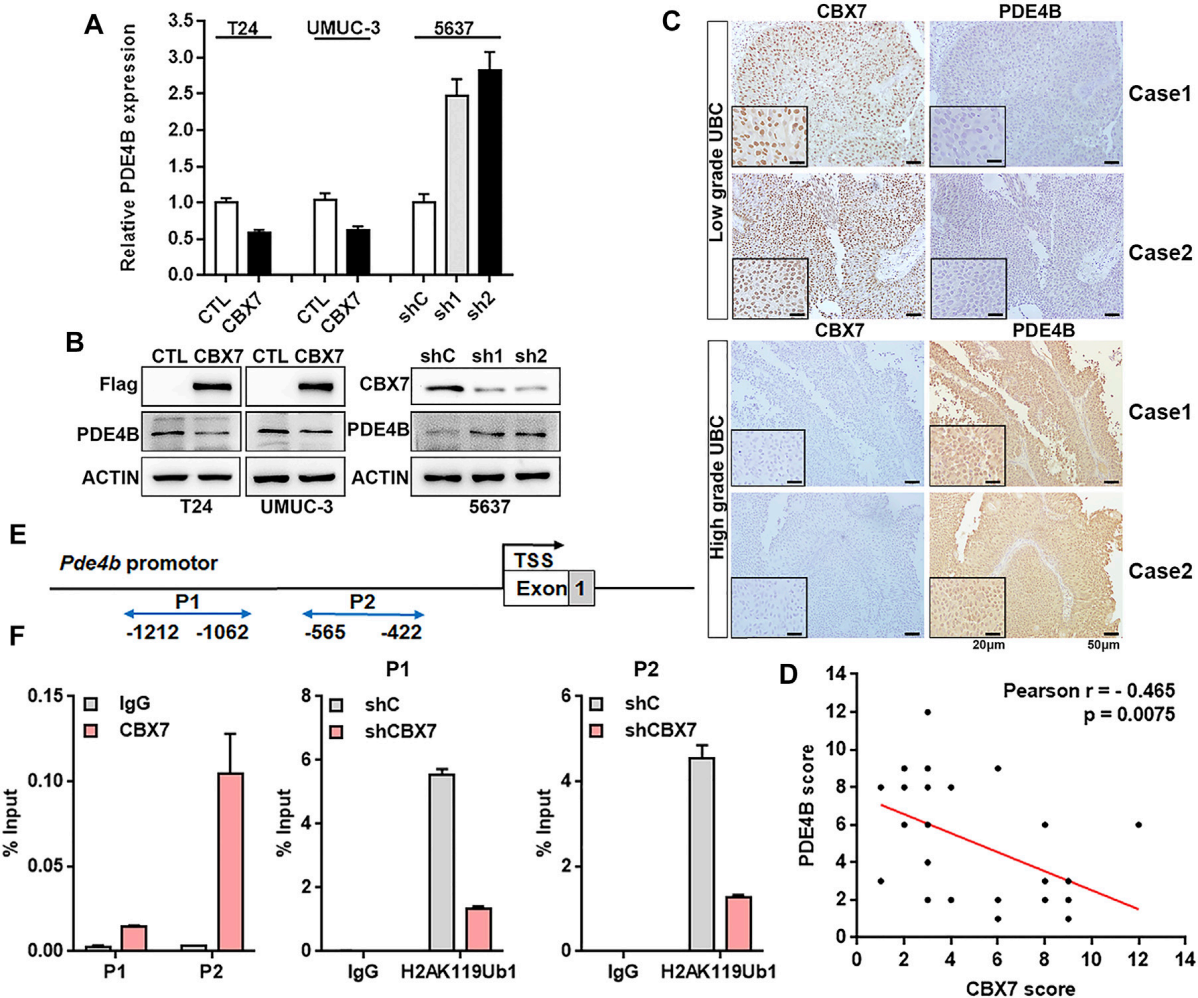
CBX7 transcriptionally represses PDE4B in a PRC1-dependent manner **(A,B)** PDE4B mRNA and protein level upon CBX7 knockdown or overexpression were measured by qRT-PCR **(A)** and western blotting **(B)**. **(C)** representative IHC staining images of CBX7 and PDE4B expression in low-grade and high-grade patients with UBC. Scale bar, 50 and 20 μm. **(D)** Pearson correlation between CBX7 and PDE4B protein expression in UBC patients from IHC cohort. **(E)** Schematic diagram of the PDE4B promoter examined by ChIP analysis. **(F)** ChIP assay analysis of CBX7 and ubH2AK119 for the PDE4B promoter region in 5,637 cells with or without CBX7 deficiency.

To confirm this hypothesis, qRT-PCR and Western blotting were applied to determine whether CBX7 can negatively regulate the expression of PDE4B. As shown in Figures 6A,B, PDE4B was decreased in CBX7-overexpressed T24 and UMUC-3 cells, but increased in CBX7-silenced 5,637 cells at both mRNA and protein levels. We further detected the association between PDE4B and CBX7 expression levels in UBC patients. The IHC data showed that CBX7 mainly expressed in the nuclei of low grade UBC cells, but weakly positive signal in high grade, while PDE4B-positive signals were barely detected in low grade UBC cells, but strong positive signals in cytosol were observed in high grade UBC cells (Figure 6C). Of note, the expression of CBX7 in UBC samples was negatively associated with the expression of PDE4B (*n* = 32, *p* = 0.0075, Figure 6D). To further determine whether CBX7 suppresses the transcription of PDE4B in a canonical PRC1-dependent manner, we carried out ChIP assays and the results revealed that CBX7 was recruited to the promoter region of PDE4B gene and consequently inhibited the PDE4B transcription by increasing the abundance of mono-ubiquitinated H2A at K119 (ubH2AK119) repressive mark at its promoter region (Figures 6E,F). Collectively, these findings indicated that PDE4B was transcriptionally repressed by CBX7 in a PRC1-dependent manner.

### PDE4B Induces UBC Cells EMT *via* β-Catenin

**FIGURE 7:**
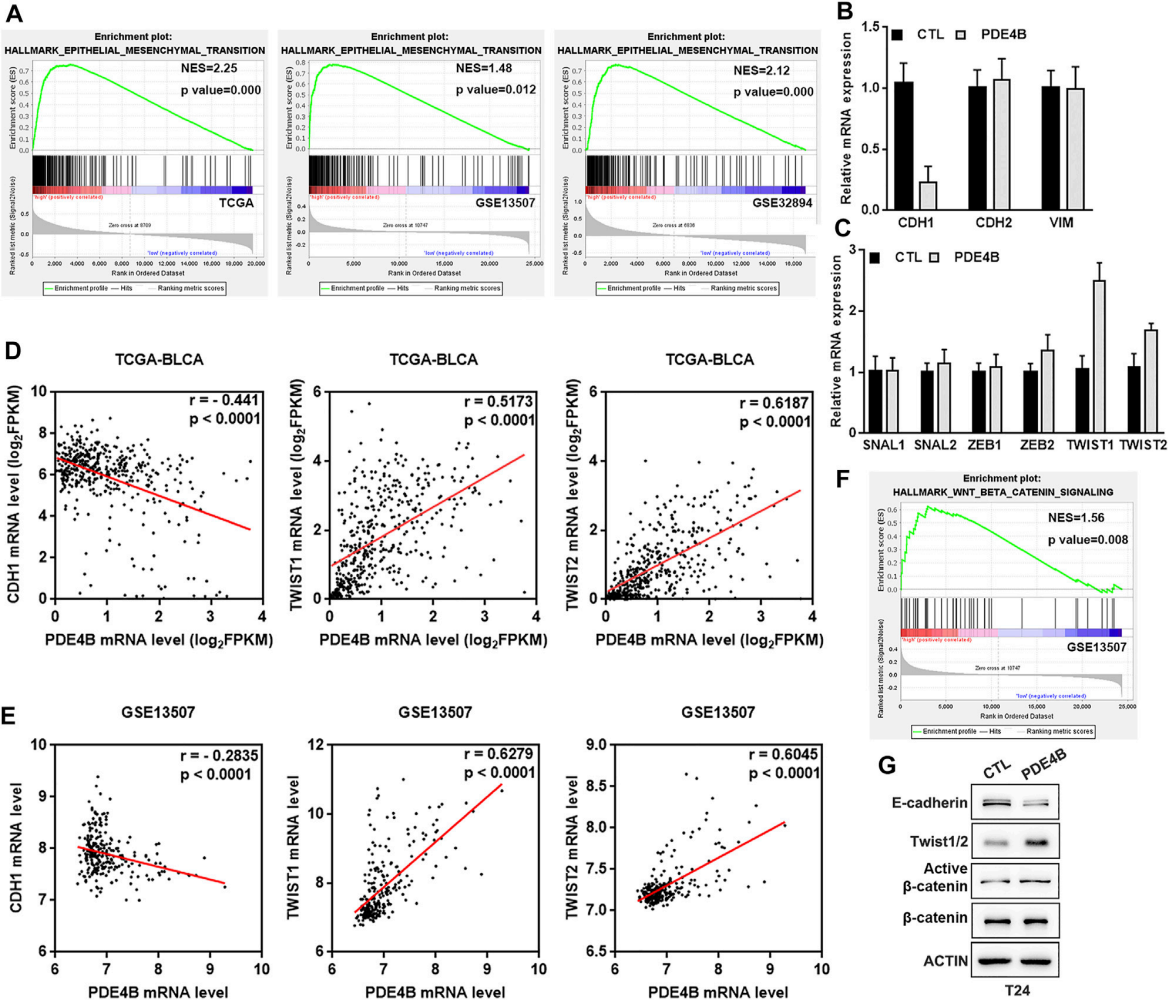
PDE4B induces UBC cells EMT *via* β-catenin pathway. **(A)** GSEA enrichment on the association of PDE4B expression with EMT gene sets based on TCGA-BLCA, GSE13507, and GSE32894 databases. **(B,C)** The mRNA expression of EMT-associated markers and transcription factors upon PDE4B overexpression were measured by qRT-PCR. **(D,E)** The correlation between PDE4B and E-cadherin, TWIST1, TWIST2 expression was analyzed by Pearson correlation analysis in UBC patients from TCGA-BLCA and GSE13507 databases. **(F)** GSEA enrichment on the association of PDE4B expression with Wnt/β-catenin pathway based on GSE13507 database. **(G)** The expression of EMT relative factors and activation of the β-catenin pathway were detected by western blotting in T24 cells with PDE4B overexpression.

With PDE4B overexpression or depletion, we observed significant changes in cell migration and invasion capabilities. These cell phenotypic changes have the same characteristics as EMT. It is universally recognized that EMT is highly correlated with tumor invasion and metastasis, in which cancer cells acquire mobility with phenotype changes (Nieto et al., 2016; Dongre and Weinberg, 2019). Thus, we suspected that EMT may be responsible for PDE4B-mediated changes in migration and invasion. As shown in Figure 7A, the GESA results revealed that high PDE4B expression was significantly correlated with EMT gene sets in TCGA-BLCA, GSE13507, and GSE32894. Accordingly, we then detected the mRNA expression of EMT-related factors, as well as transcription regulators in PDE4B-overexpressing T24 cells. The results showed that the epithelial marker E-cadherin was decreased upon PDE4B overexpression (Figure 7B). Moreover, ectopic PDE4B increased the expression of two EMT-related transcription factors TWIST1 and TWIST2 (Figure 7C). Next, we analyzed the correlation between PDE4B and E-cadherin, TWIST1 and TWIST2 in TCGA-BLCA and GSE13507 databases. Consistent with our findings, PDE4B expression showed significantly negative correlation with E-cadherin expression, but notably positive correlation with TWIST1 and TWIST2 expression (Figures 7D,E). Western blotting further proved the reduction of E-cadherin and the elevation of Twist1/2 upon PDE4B overexpression (Figure 7G). Consequently, these results revealed the role of PDE4B in EMT induction.

As a pivotal mediator in Wnt/β-catenin signaling pathway, β-catenin exerts a pivotal role in tumor invasion and metastasis (Nusse and Clevers, 2017; Zhang and Wang, 2020). Furthermore, increased expression of β-catenin could induce EMT (Yang et al., 2017; Zhu et al., 2020). To better understand the mechanism of PDE4B-mediated EMT, we performed GSEA in GSE13507 database and drew conclusion that high PDE4B expression group was enriched in the Wnt/β-catenin pathway (Figure 7F). Consistently, with PDE4B overexpression, the protein levels of active β-catenin in T24 cells were upregulated (Figure 7G). Collectively, these data implied that PDE4B modulated EMT by targeting the β-catenin signaling.

### Rolipram Suppresses UBC Invasion *via* Inhibiting the PDE4B

**FIGURE 8:**
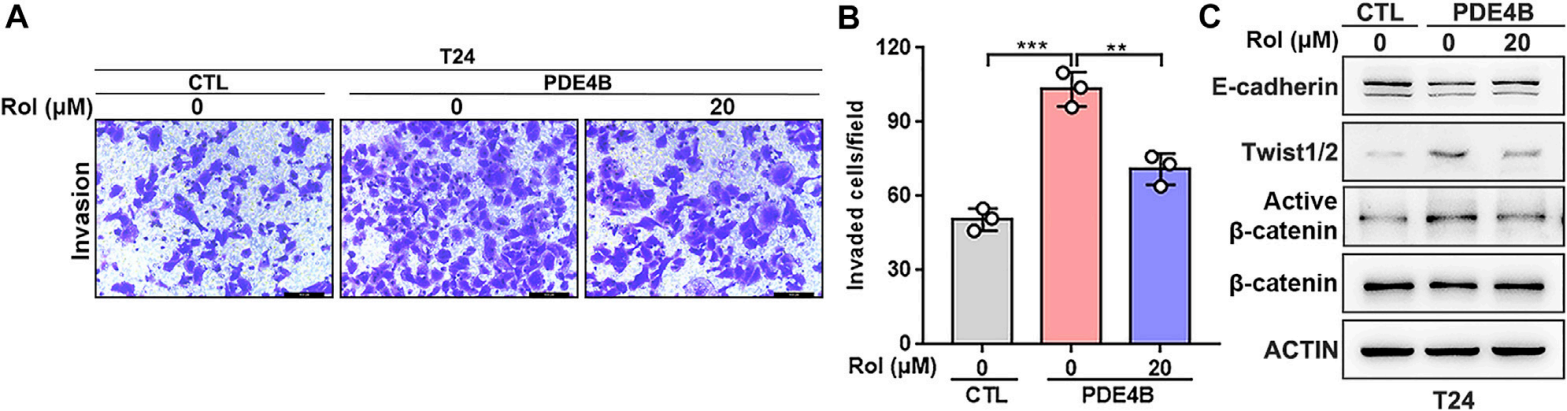
Rolipram suppresses UBC invasion *via* inhibiting the PDE4B activity. **(A,B)** PDE4B overexpression enhanced cell invasive ability, whereas this effect was partly abolished by Rolipram (Rol) at dose of 20 μM. Scale bar, 100 μm. **(C)** Gene expression was examined by western blotting in PDE4B-overexpressing cells after treated with Rol at dose of 20 μM **p < 0.01, ***p < 0.001.

Next, we investigated the potential therapeutic value of PDE4B in UBC. Rolipram (Rol) was reported to be an effective PDE4B inhibitor, which exerted important tumor suppressive effects by inhibiting the PDE4B/mTOR/Myc axis in colorectal cancer (Kim et al., 2019). Thus, we explored whether pharmacological inhibition of PDE4B by rolipram could rescue the tumor-promoting effects induced by PDE4B overexpression. The results of invasion assays showed that rolipram at 20 μM significantly reduced the number of invaded cells in PDE4B-overexpressing T24 cells (Figures 8A,B). Consistently, treatment with rolipram partly rescued the protein level of E-cadherin, Twist1/2 and β-catenin (Figure 8C). Altogether, we concluded that pharmacological repression of PDE4B by rolipram could partly rescue the tumor-promoting effects induced by PDE4B overexpression.

## Discussion

PDE4 enzymes are divided into four subtypes (PDE4A, 4B, 4C, and 4D) (Conti et al., 2003). Currently, promising treatments are being developed for neurological diseases that against PDE4B and PDE4D (Fox et al., 2014; Hagen et al., 2014; Zhang et al., 2017). PDE4 family inhibitors are effective inflammation inhibitors, which have been licensed to treat the inflammatory diseases ranging from chronic obstructive pulmonary disease (COPD) to arthritis (Wittmann and Helliwell, 2013; Li et al., 2018; Phillips, 2020). The main challenge of PDE4s inhibitors is their narrow therapeutic index and the nausea, vomiting and other side effects also limit the gastrointestinal tolerability. Given that PDE4 is such a promising clinical target, increasing studies have continued to seek strategies to expand its therapeutic index.

In this study, we reported PDE4B as a promising prognostic and therapeutic target in UBC. Dysregulation of PDE4B has been reported in a variety of cancers, but its clinical significance has not yet been elucidated. Previous studies reported that PDE4B expression was increased in diffuse large B-cell lymphoma (Smith et al., 2005) and non-small cell lung cancer (He et al., 2017), but was downregulated in prostate cancer (Kashiwagi et al., 2012). In our study, we detected that most UBC cell lines expressed higher level of PDE4B expression compared with nonmalignant urothelial cell line. Besides, we also observed that higher expression of PDE4B was specifically in basal/squamous subtype, which was the UBC subtypes with high malignant tumor grade and strong metastasis. Moreover, in TCGA-BLCA and GEO cohorts, patients with high PDE4B expression experienced aggressive clinicopathological characteristics and unfavorable prognosis, compared with those low PDE4B expression. Our data from UBC samples also draw the same conclusion. Accordingly, we proposed the oncogenic role of PDE4B in UBC progression. With gain- and loss-of-function assays, we demonstrated that PDE4B facilitated UBC cell proliferation, migration and invasion. We further identified CBX7 as a regulator of PDE4B to inhibit the expression of PDE4B at the transcription level in a PRC1-dependent manner. Previous research also demonstrated that CBX7 inhibited the malignant progression of UBC by means of transcription repression (Huang et al., 2021b). Taken together, these findings suggested that CBX7-mediated transcriptional repression regulatory network exerted significant inhibitory effect in the progression of UBC.

Accumulating studies have emphasized the significance of EMT in driving cancer development from occurrence to metastasis, in which cells acquire exercise capacity with phenotype changes, such as apical-basal polarity and adhesion junction (Lu and Kang, 2019; Pastushenko and Blanpain, 2019). The process of EMT is sparked by multiple extracellular signals, including growth factors and extracellular matrix components, and is mediated by the activation of EMT transcription factors (De Craene and Berx, 2013; Lamouille et al., 2014). In this study, we confirmed that PDE4B expression was strongly associated with EMT gene sets. Our results showed that PDE4B overexpression reduced the expression of epithelial markers, but enhanced the expression of transcription factors Twist1/2. These results indicated that the effects of PDE4B on cell migration and invasion were related to the EMT process. Consistently, previous study identified TEAD4 as a prognostic marker in UBC for promoting cell migration and invasion *via* EMT through activating the expression of Twist1/2 (Huang et al., 2021a). Collectively, these researches implied that the EMT activated by TWIST plays an important role in the progression of UBC, suggesting that TWIST is a promising therapeutic target against UBC. It has been universally acknowledged that Wnt/β-catenin signaling could induce the EMT process and downregulate the expression of E-cadherin (Nelson and Nusse, 2004; Fagotto, 2013). Therefore, we proposed a possibility, based on our experimental results, that PDE4B-induced EMT is achieved by activating Wnt/β-catenin signaling. However, further study should be performed to investigate the specific association among PDE4B, EMT and Wnt/β-catenin signaling pathway in UBC.

Previous study has demonstrated that rolipram was an effective PDE4B inhibitor, which exerted important tumor suppressive effects by inhibiting the PDE4B/mTOR/Myc axis in colorectal cancer (Kim et al., 2019). Our data consistently revealed that inhibition of PDE4B by rolipram could rescue the tumor-promoting effects from PDE4B overexpression on cell invasion and EMT, suggesting that rolipram would be a promising UBC pharmacological inhibitor targeting PDE4B.

Taken together, our current *in vitro* findings support the notion that PDE4B acts as an oncogene in UBC. Hence, the further investigation on its *in vivo* role is guaranteed. We will explore the effects of PDE4B at the animal level in the following research. Recently, the studies on PDE4B knock-out mice have been clearly evidenced. First, PDE4B^−/−^ mice exhibited no overt morphological abnormalities, similar body weight, growth rate and litter size, compared to those of wild-type littermates (Jin and Conti, 2002). Moreover, two research groups reported that PDE4B deficiency reduced lung injury and alcohol-induced brain inflammation in mice, respectively (Avila et al., 2017; Dhar et al., 2021). Since our finding also revealed its tumor promoting role of PDE4B and inhibition of its activity can reverse its oncogenic function. Taken together, PDE4B may be a promising therapeutic target.

In summary, we reported PDE4B is transcriptionally suppressed by CBX7 and acts as an oncogene with prognostic significance in UBC. Our results indicated that PDE4B reinforced UBC cell migration and invasion through inducing EMT mediated by β-catenin. In view of the development and progress of compounds that inhibit the function of PDE4 family proteins, our research provides a novel therapeutic target for the treatment of UBC.

## Data Availability

The datasets presented in this study can be found in online repositories. The names of the repository/repositories and accession number(s) can be found in the article/Supplementary Material.
