# Supplementary material for: PDE4B Induces Epithelial-to-Mesenchymal Transition in Bladder Cancer Cells and Is Transcriptionally Suppressed by CBX7
Source: Front Cell Dev Biol. 2021 Dec 16;9:783050. doi: 10.3389/fcell.2021.783050 (PMC8716816; doi:10.3389/fcell.2021.783050)
Supplement: Supplementary file 1 [file DataSheet1.docx]

Supplementary Materials

# Supplementary Tables

| **Items** | **Company** | **Cat. No.** | |
| --- | --- | --- | --- |
| **Reagents and Chemicals** |  | |  |
| ChIP assay kit | Merck Millipore | | #17-371 |
| Crystal violet | Sangon Biotech | | A100528 |
| MTT | Beyotime | | ST316 |
| Matrigel gel | BD Biosciences | | 356230 |
| Prime-Script RT-PCR kit | TaKaRa | | RR047A |
| Puromycin | Yeason | | ISY1130 |
| Rolipram | MedChemExpress | | HY-16900 |
| TRIzol reagent | TaKaRa | | 9109 |

**SUPPLEMENTARY TABLE 1** | List of reagents and chemicals.

**SUPPLEMENTARY TABLE 2** | List for sequences of primer sets, shRNA and siRNAs

|  | **Direction** | **Sequences (5’-3’)** |
| --- | --- | --- |
| **Primer sets for qRT-PCR** |  |  |
| *β-actin* | Forward | CATGTACGTTGCTATCCAGGC |
|  | Reverse | CTCCTTAATGTCACGCACGAT |
| *CBX7* | Forward | GCGTGCGGAAGGGTAAAGT |
|  | Reverse | GCTTGGGTTTCGGACCTCTC |
| *CDH1* | Forward | AATAGTGCCTAAAGTGCTGC |
|  | Reverse | AGACCCACCTCAATCATCCT |
| *CDH2* | Forward | AGCCAACCTTAACTGAGGAGT |
|  | Reverse | GGCAAGTTGATTGGAGGGATG |
| *PDE4B* | Forward | CAAGCATCTCACGCTTTGGAG |
|  | Reverse | GCCACGTCAGAATGGTAATGG |
| *SNAI1* | Forward | TCGGAAGCCTAACTACAGCGA |
|  | Reverse | AGATGAGCATTGGCAGCGAG |
| *SNAI2* | Forward | CTGGGCGCCCTGAAGATGCAT |
|  | Reverse | GGCTTCTCCCCCGTGTGAGTTCTA |
| *TWIST1* | Forward | CACGAGCGGCTCAGCTACGC |
|  | Reverse | ACAATGACATCTAGGTCTCCGGCCC |
| *TWIST2* | Forward | AGTCGAGCGAAGATGGCAG |
|  | Reverse | GGAAGTCTATGTACCTGGCGG |
| *VIM* | Forward | AGTCCACTGAGTACCGGAGAC |
|  | Reverse | CATTTCACGCATCTGGCGTTC |
| *ZEB1* | Forward | TTACACCTTTGCATACAGAACCC |
|  | Reverse | TTTACGATTACACCCAGACTGC |
| *ZEB2* | Forward | GACAGATCAGCACCAAATGC |
|  | Reverse | GCTGATGTGCGAACTGTAGG |
| **Primer sets for ChIP analysis** | | |
| PDE4B promoter-P1F |  | GTGCACAGTAGGTGTGCATGA |
| PDE4B promoter-P1R |  | AGCTGGCAAGGAAAAGAGGAG |
| PDE4B promoter-P2F |  | GATGCACAGCGAGTGACTGA |
| PDE4B promoter-P2R |  | CCTGGTTTGAACCCCACACT |
| **PCR for shRNA subcloning** |  |  |
| shCBX7-1F |  | CCGGGCCAGAAGAGCACATCTTGTTCAAGAGA  CAAGATGTGCTCTTCTGGCTTTTTG |
| shCBX7-1R |  | AATTCAAAAAAGCCAGAAGAGCACATCTTG  TCTCTTGAACAAGATGTGCTCTTCTGGC |
| shCBX7-2F |  | CCGGTCATGGCCTACGAGGAGAATTCAAGAGA  TTCTCCTCGTAGGCCATGATTTTTG |
| shCBX7-2R |  | AATTCAAAAAATCATGGCCTACGAGGAGAA  TCTCTTGAATTCTCCTCGTAGGCCATGA |
| **siRNA sequences** |  |  |
| siNC |  | UUCUCCGAACGUGUCACGUTT |
| siPDE4B-1 |  | CUGAUGAUAAUGUUAAAGATT |
| siPDE4B-2 |  | GCUGGAUAUUCUCACAAUATT |

NC, negative control.

**SUPPLEMENTARY TABLE 3** | List of antibodies

| **Antibody** | **Company** | **Cat. No.** | **Dilution (Application)** |
| --- | --- | --- | --- |
| ACTIN | Abclonal | AC206 | 1:2,000 (WB) |
| Anti-Flag | Abclonal | AE-005 | 1:1,000 (WB) |
| Active β-catenin | Cell Signaling Technology | 8814S | 1:1,000 (WB) |
| β-catenin | Cell Signaling Technology | 9581S | 1:1,000 (WB) |
| CBX7 | Abcam | ab21873 | 1:1,000 (WB) |
| E-Cadherin | Proteintech | 20874-1-AP | 1:1,000 (WB) |
| ubH2AK119 | Cell Signaling Technology | 8240S | 1:1,000 (WB) |
| PDE4B | Abcam | ab14611 | 1:500 (WB, IHC) |
| TWIST1/2 | GeneTex | GTX108750 | 1:1,000 (WB) |
| 2nd antibody mouse IgG | Cell Signaling Technology | 7076s | 1:5,000 (WB) |
| 2nd antibody rabbit IgG | Cell Signaling Technology | 7074s | 1:5,000 (WB) |

# Supplementary Figures

**SUPPLEMENTARY FIGURE 1**


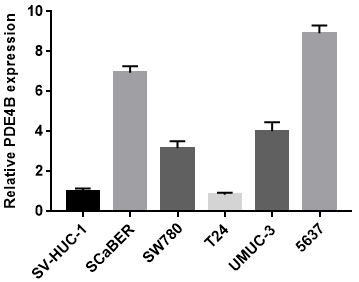


**SUPPLEMENTARY FIGURE 1** | Endogenous expression of PDE4B in multiple cell lines were detected by qRT-PCR.

**SUPPLEMENTARY FIGURE 2**


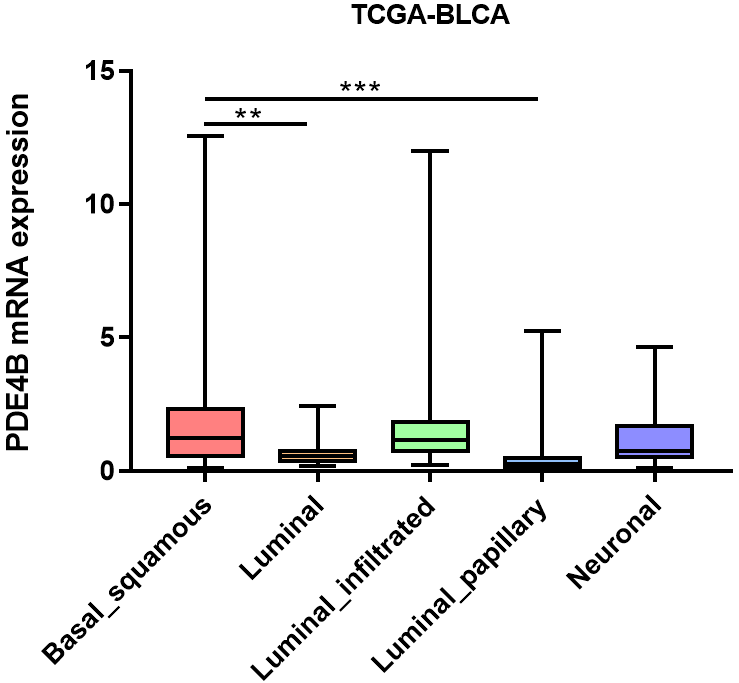


**SUPPLEMENTARY FIGURE 2** | PDE4B mRNA expression in five UBC subtypes.
